# Challenging the curve: can ChatGPT-generated MCQs reduce grade inflation in pharmacy education

**DOI:** 10.3389/fphar.2025.1516381

**Published:** 2025-01-29

**Authors:** Dalia Almaghaslah

**Affiliations:** Department of Clinical Pharmacy, College of Pharmacy, King Khalid University, Abha, Saudi Arabia

**Keywords:** AI, ChatGPT4, MCQ, pharmacy course, grade inflation, AI-generated MCQs

## Abstract

**Introduction:**

Grade inflation in higher education poses challenges to maintaining academic standards, particularly in pharmacy education, where assessing student competency is crucial. This study investigates the impact of AI-generated multiple-choice questions (MCQs) on exam difficulty and reliability in a pharmacy management course at a Saudi university.

**Methods:**

A quasi-experimental design compared the 2024 midterm exam, featuring ChatGPT-generated MCQs, with the 2023 exam that utilized human-generated questions. Both exams covered identical topics. Exam reliability was assessed using the Kuder-Richardson Formula 20 (KR-20), while difficulty and discrimination indices were analyzed. Statistical tests, including t-tests and chi-square tests, were conducted to compare performance metrics.

**Results:**

The 2024 exam demonstrated higher reliability (KR-20 = 0.83) compared to the 2023 exam (KR-20 = 0.78). The 2024 exam included a greater proportion of moderate questions (30%) and one difficult question (3.3%), whereas the 2023 exam had 93.3% easy questions. The mean student score was significantly lower in 2024 (17.75 vs. 21.53, p < 0.001), and the discrimination index improved (0.35 vs. 0.25, p = 0.007), indicating enhanced differentiation between students.

**Discussion:**

The findings suggest that AI-generated MCQs contribute to improved exam rigor and a potential reduction in grade inflation. However, careful review of AI-generated content remains essential to ensure alignment with course objectives and accuracy.

**Conclusion:**

AI tools like ChatGPT offer promising opportunities to enhance assessment integrity and support fairer evaluations in pharmacy education.

## 1 Introduction

The average GPA (Grade Point Average) of university students has been steadily increasing over the last 40 years. While this trend has been linked to various factors, including advancements in teaching methods, modern educational technologies, and the implementation of quality standards, it is also associated with grade inflation (Maamari and Naccache, 2022; Carter and Lara, 2016).

Grade inflation is defined as “an increase in GPA without a concomitant increase in achievement.” Another explanation describes it as “students receiving higher grades regardless of actual academic attainment.” Simply put, grade inflation means students are awarded higher grades without demonstrating enhanced levels of mastery (Chowdhury, 2018).

Grade inflation has become normalized in various academic institutions worldwide. It is a phenomenon observed in schools, colleges, and universities in countries such as the USA, UK, Canada, France, and Sweden. Educators have noted that current students often receive higher grades for the same quality of work submitted by their predecessors. This trend is evident across higher education fields, including professional education programs such as pharmacy (Chowdhury, 2018).

Grade inflation was reported in pharmacy education (Cain et al., 2012). A study reported grade inflation in pharmacy science courses, Pharmacy practice courses, and experiential training at a rate of 1% each year over the study period of 20 years (Granberry and Stiegler, 2003).

The causes of grade inflation are varied, with teachers being primary contributors. Teacher grading practices are closely linked to their instructional perspectives. Performance-oriented teachers often emphasize competence-based assessments, which are typically reflected in grades. They may implement less challenging assessments that make it easier for students to achieve higher grades. Conversely, learning-oriented teachers adopt approaches that promote deeper understanding and comprehension. Their orientation involves more complex tasks and assessment methodologies, focusing on knowledge acquisition rather than merely obtaining high grades (Granberry and Stiegler, 2003; Arsyad Arrafii, 2020).

Additionally, other instructor-related factors contribute to grade inflation, such as student evaluations of instructors, which are often used to gauge teaching effectiveness and serve as a basis for contract renewals, awards, and promotions. In pursuit of job and financial security, some educators may practice grade inflation to receive favorable evaluations and secure their positions. Furthermore, some teachers may inflate grades out of concern for students’ psychological wellbeing or fear that lower grades could harm students’ prospects in a competitive job market after graduation (Chowdhury, 2018).

Institutional factors also play a significant role in grade inflation. Many academic institutions have become enrollment-oriented, aiming to attract and retain more students. To achieve this, they may prioritize student satisfaction, which can sometimes be facilitated through grade inflation. By ensuring students receive higher grades, institutions may enhance their reputation and public image, attract a larger student body, and improve retention rates. This emphasis on maintaining a positive public image and strong reputation further incentivizes the practice of grade inflation (Chowdhury, 2018).

Recently, the use of artificial intelligence (AI) in education has expanded, particularly in medical education. ChatGPT, a large language model (LLM) developed by OpenAI, is one example. These LLMs are trained on massive datasets and can generate human-like text with high accuracy by utilizing complex mathematical models and parameters (Cheung et al., 2023).

AI tools like ChatGPT have been employed in medical education for various purposes, including teaching, grading, providing feedback, and assisting in designing lectures and classes. It has also been used to facilitate frequent assessments and continuous evaluations through the creation of assessment tasks. Specifically, AI like ChatGPT has been used to generate multiple-choice questions (MCQs) to ensure rigorous and consistent evaluations in education. (Kyung, 2024; Ngo et al., 2024).

This combination of AI-driven assessments and more stringent grading practices may offer a solution to grade inflation by ensuring students are evaluated based on deeper cognitive skills and complex tasks, reducing the frequency of inflated grades.

This combination of AI-driven assessments and more stringent grading practices may offer a solution to grade inflation by ensuring students are evaluated based on deeper cognitive skills and complex tasks, reducing the frequency of inflated grades.

In the context of a Saudi college of pharmacy, a pharmacy management course—a 3-credit course within the PharmD program—had shown grade inflation for two consecutive terms. This trend highlighted the need for more comprehensive and rigorous assessment methods. In this educational setting, students tended to view assignments as almost guaranteed full marks, with written exams being more culturally accepted as a means of objectively controlling the difficulty level.

To address the grade inflation issue, the course incorporated ChatGPT to create deeper-learning assessment questions, specifically through the generation of multiple-choice questions (MCQs). By utilizing AI, the aim was to enhance the complexity and rigor of assessments, encouraging students to engage in more critical thinking and deeper understanding, rather than relying on surface-level learning. This shift toward AI-generated assessments was intended to balance grading practices and improve the evaluation of student knowledge in a more objective and culturally appropriate manner.

The study was conducted to assess whether the integration of ChatGPT for generating more challenging MCQs could help indirectly combat grade inflation. By creating assessments that require deeper understanding and critical engagement, the research aimed to determine if these AI-generated questions led to a reduction in inflated grades, promoting a more accurate evaluation of students’ knowledge and skills in the pharmacy management course.

## 2 Methodology

### 2.1 Study design

This quasi-experimental study was conducted during the midterm exam of 2024 in a 3-credit pharmacy management course within a PharmD program at a Saudi college of pharmacy. The study aimed to determine whether ChatGPT-generated MCQs could combat grade inflation by increasing exam difficulty and whether the AI-generated questions aligned with human-generated assessments in terms of difficulty. The findings from the 2024 AI-generated MCQ midterm exam were compared with results from the 2023 human-generated MCQ exam to evaluate any differences in assessment performance and grade inflation. Table 1 shows the course assessment structure. The course assessment structure is summarized in Table 1.

**TABLE 1:** Course assessment structure.

Course assessments		Marks
Assignment I		15
Midterm exam	30 MCQs^a^	24
	3 essays	6
Assignment II		15
Final exam	50 MCQs	40
Total		100

^a^ The study intervention.

### 2.2 Participants

A total of 57 students enrolled in the course participated in the midterm exam (2024), which featured ChatGPT-generated and instructor-reviewed MCQs, along with essay questions.

### 2.3 ChatGPT question generation and selection process

The course textbook chapters covering six topics were uploaded to ChatGPT-4o one at a time, and the AI was instructed to generate 20 difficult MCQs per topic. A panel of three course instructors reviewed and evaluated these questions based on the following criteria:

1. Appropriateness: Alignment with course objectives and well-constructed design.
2. Clarity and Specificity: Free of ambiguity and clearly stated.
3. Relevance: Consistent with the course content and clinical context.
4. Discriminative Power: Ability of the distractors to differentiate between high and low performers.
5. Graduate-Level Suitability: Encouragement of higher-order thinking in accordance with Bloom’s taxonomy.

From the reviewed questions, the instructors selected a total of 30 MCQs for the midterm exam, five questions from each topic. Each topic featured two difficult questions and three easy or moderate questions. Each MCQ was worth 0.8 points, contributing to a total of 24 points for the MCQ section. Additionally, the exam included three essay questions, each worth 2 points, adding 6 points to the total midterm exam score.

### 2.4 Data analysis

The MCQs were analyzed using the Difficulty Index (DIF) and Discrimination Index (DI) to evaluate the quality of the items. The internal reliability of the test scores was assessed using the Kuder-Richardson Formula 20 (KR-20), providing a measure of test consistency. The scores were analyzed based on proportions, means, standard deviations, correlation statistics, and t-tests to determine item discrimination and difficulty.

Student performance was ranked, and the top one-third of students were categorized as high achievers, while the bottom one-third were identified as low achievers. This classification enabled a more detailed analysis of the test items’ ability to differentiate student performance.

### 2.5 Difficulty index (DIF)

The DIF was calculated using the formula:

DIF=H+L/N

Where:

• H = Number of high-achieving students answering the item correctly
• L = Number of low-achieving students answering the item correctly
• N = Total number of students in both groups

A DIF score below 30% indicates a very difficult item, while a score above 70% denotes an easy item. Scores between 30% and 70% are considered acceptable. The DIF and DI are inversely related (Al Ameer, 2023).

### 2.6 Discrimination index (DI)

The DI was calculated using the following formula:

DI=H‐L/N*2

The DI measures the ability of an item to distinguish between high- and low-achieving students. The DI values were interpreted as follows:

• Negative DI: Poor discrimination
• 0.19 or less: Poor discrimination
• 0.2 to 0.29: Acceptable discrimination
• 0.3 to 0.39: Good discrimination
• ≥ 0.4: Excellent discrimination

Higher DI values indicate better discrimination between student groups (Al Ameer, 2023).

### 2.7 Ethical considerations

An ethical clearance was given by the Ethics Committee at King Khalid university ECM#2024-3123.

## 3 Results

Questions with scores of less than 30% correct responses were considered difficult. In the 30-item MCQ from the 2024 exam, there was 1 difficult question (3.3%), compared to no difficult questions in 2023. The number of moderate questions, with scores between 30%–70%, increased from 2 (6.7%) in 2023 to 9 (30%) in 2024. The total number of easy questions, defined as those with scores greater than 70%, decreased from 28 (93.3%) in 2023 to 20 (66.7%) in 2024. These results suggest a shift towards more moderate-difficulty questions in 2024, indicating an improvement in the balance of question difficulty compared to 2023. The difference in student numbers between 2023 and 2024 attributed to variations in enrollment during these academic years (Table 2).

**TABLE 2:** Psychometric measurements.

Variable	2023	2024
Number of questions	30	30
Number of students	57	59
Number of difficult questions	0	1
Number of moderate questions	2	9
Number of easy questions	28	20
Mean test score out of 24 marks	21.52982	17.74915
Range of test score	12.0–24.0	4.0–23.2
KR-20 reliability	0.78	0.83

The KR-20 reliability coefficients for the exams were calculated as 0.78 for 2023 and 0.83 for 2024, both of which fall within an acceptable range, indicating good internal consistency. A KR-20 value above 0.7 is generally considered acceptable, suggesting that the questions in both exams consistently measured the intended construct.

The slightly higher reliability in 2024 (0.83) implies an improvement in the alignment, clarity, or quality of the questions compared to 2023 (0.78). This increase may reflect efforts to refine the exam content, potentially reducing ambiguity or enhancing the relevance of the questions. Overall, both exams demonstrate solid internal consistency, with the 2024 exam showing marginally better reliability, which may contribute to more valid assessments of student knowledge and performance.

### 3.1 Midterm exam scores

The mean score for the 2023 exam was 21.53 ± 2.51, while for the 2024 exam it was 17.75 ± 3.88. A two-sample t-test revealed a statistically significant difference between the 2 years (t = 6.21, p < 0.001).

The lower mean score and higher standard deviation in 2024 suggest that the exam was more challenging, leading to greater variability in student performance and potentially reduced grade inflation (Table 3).

**TABLE 3:** Comparison of midterm exam scores, difficulty, and discrimination indices between 2023 and 2024 pharmacy management exams.

	Mean	Sd	Test statistic	P value
Midterm exam score/24				
2023	21.5298456	2.51	6.210841548	8.83E-09
2024	17.74915254	3.88		
Difficulty index				
2023	0.904678	0.113012	3.689935	0.000497
2024	0.75189	0.196632		
Discrimination Index				
2023	0.25	0.05	2.85	0.007
2024	0.35	0.1		

### 3.2 Difficulty index

The mean difficulty index for the 2023 exam was 0.905 ± 0.113, compared to 0.752 ± 0.197 in 2024.

The t-test for difficulty index also showed a significant difference (t = 3.69, p = 0.000497), indicating that questions in 2024 were more difficult on average.

The shift in difficulty suggests that the 2024 exam was designed with more challenging questions, better differentiating between student abilities (Table 3).

### 3.3 Discrimination index

The mean discrimination index improved from 0.25 ± 0.05 in 2023 to 0.35 ± 0.10 in 2024.

The t-test revealed a significant increase in discrimination (t = 2.85, p = 0.007), suggesting that the 2024 exam was more effective in distinguishing between high- and low-performing students.

Importantly, the discrimination indices for both exams are acceptable, as they are higher than 0.1, indicating that the questions were able to distinguish adequately between students of varying performance levels (Table 3).

To assess whether the students’ grades followed a normal distribution across 2023 and 2024, a Shapiro-Wilk test for normality was conducted, skewness was examined, histograms were plotted, and an Anderson-Darling test was performed to evaluate differences between the two distributions.

The Shapiro-Wilk test for normality indicated that the grades for both 2023 and 2024 significantly deviate from a normal distribution. The 2023 grades had a Shapiro-Wilk statistic of 0.80 and a p-value of 7.24 × 10⁻^7^, while the 2024 grades showed a statistic of 0.919 and a p-value of 0.00081, both falling below the 0.05 threshold for normality. These results confirm that neither year’s grades follow a normal distribution. Additionally, the skewness values highlight a negative skew in both years, with the 2023 grades showing a skewness of −1.77, indicating a highly left-skewed distribution with grades concentrated at the higher end. In comparison, the 2024 grades exhibited a skewness of −1.08, reflecting a moderately left-skewed distribution. This reduction in skewness suggests that the distribution of grades in 2024 was more balanced, with fewer students receiving extremely high scores. Overall, the shift toward a less skewed distribution in 2024 may indicate a positive change toward mitigating grade inflation and achieving a more equitable assessment outcome (Table 4).

**TABLE 4:** Normality and skewness analysis of midterm exam scores for 2023 and 2024.

Year	Shapiro-wilk statistic	Shapiro-wilk p-value	Skewness
2023	0.80	7.24 × 10⁻^7^	−1.77
2024	0.919	0.00081	−1.08

Anderson-Darling test revealed a statistic of 26.84 with a p-value <0.001, indicating a statistically significant difference between the grade distributions of 2023 and 2024. This result suggests that the two distributions are not only distinct but also differ significantly in their shape, variability, or overall patterns. The significant difference aligns with the earlier findings of skewness reduction in 2024, indicating that the distribution of grades shifted toward a more balanced pattern compared to 2023. These results may reflect the impact of changes in the assessment design, potentially contributing to a decrease in grade inflation and improved fairness in student evaluations.

#### 3.3.1 Histograms of grade distributions

**FIGURE 1:**
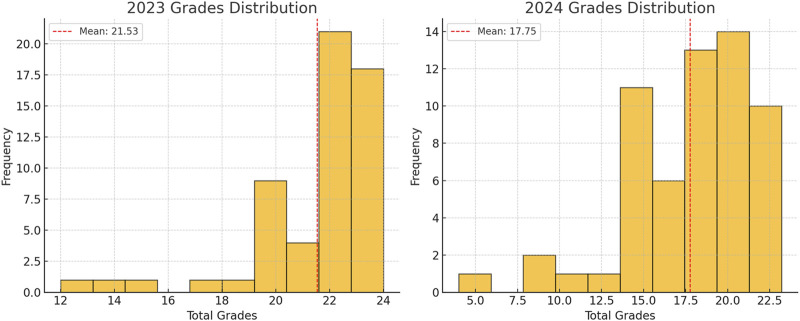
Comparison of grade distributions in the 2023 and 2024 pharmacy management midterm exams.

The histograms below visually confirm the skewness for both years, showing a clustering of grades toward higher scores with a few lower grades extending the tail to the left (Figure 1).

## 4 Discussion

This study explored the impact of AI-generated multiple-choice questions (MCQs) on the quality of assessments in pharmacy education by comparing exam results from 2023 to 2024. The primary objective was to determine whether the inclusion of more balanced and challenging questions, developed with the aid of ChatGPT, could reduce grade inflation and enhance the reliability of student evaluations. Several key findings were observed, including shifts in the distribution of student grades, improvements in test reliability, and adjustments in the difficulty of exam questions. These findings suggest that AI-assisted question generation can contribute to a more effective assessment framework, consistent with emerging research on the role of AI in education.

The Shapiro-Wilk test for normality revealed that the total scores for both 2023 and 2024 deviated significantly from a normal distribution (p < 0.05). However, the 2024 distribution was closer to normal (statistic = 0.92) compared to 2023 (statistic = 0.79). This shift reflects the reduced skewness observed in 2024 (skewness = −1.08), compared to the more pronounced left-skewness in 2023 (skewness = −1.77). The 2023 exam exhibited a strong clustering of high scores, suggesting that the assessment may have been too easy, contributing to grade inflation. In contrast, the more moderate skewness in 2024 indicates that the exam results were more evenly distributed, potentially reflecting a more accurate differentiation of student abilities.

Further supporting this observation, the KR-20 reliability coefficients for both years indicate solid internal consistency, with a slight improvement in 2024 (KR-20 = 0.83) over 2023 (KR-20 = 0.78). KR-20 values above 0.7 are considered acceptable, suggesting that both exams effectively measured the intended learning outcomes. The improvement in reliability in 2024 reflects a higher alignment of questions with the course objectives, likely due to the incorporation of AI-generated questions designed to address various levels of difficulty. Reliable assessments are essential in higher education to ensure that students are fairly evaluated, and this improvement indicates that the AI-assisted approach helped maintain or enhance the quality of the exam.

The analysis of the difficulty index (p-value) highlights a key difference between the two exams. The 2023 exam had an overall difficulty index of 0.90, indicating that most questions were relatively easy, with many questions having indices near or at 1.00. In contrast, the overall difficulty index for 2024 was 0.75, suggesting a more balanced distribution of easy, moderate, and challenging questions. This shift toward moderate difficulty likely contributed to the reduction in grade inflation, as students were more thoroughly evaluated across a range of competencies. Notably, several questions in 2024 had difficulty indices below 0.50, such as Q12 (0.27) and Q8 (0.37), which indicate that these questions posed a significant challenge to students. The presence of such challenging questions, along with easier ones, helps create a more robust assessment that distinguishes between different levels of student performance.

Previous studies have reported results comparable to the findings of the current study in terms of difficulty and discrimination indices for MCQ-based assessments. Kiyak et al. (2024) evaluated two case-based scenarios, each with 25 MCQs, achieving discrimination indices of 0.41 and 0.39, both exceeding the ideal threshold of 0.3. The corresponding difficulty indices were 0.78 and 0.58, indicating easy and moderate levels of difficulty, respectively (Kiyak et al., 2024). Similarly, Laupichler et al. (2024) reported a discrimination index of 0.24 for 21 MCQs, meeting the acceptable benchmark of 0.2, along with an average difficulty index of 0.62 (Laupichler et al., 2024). Zuckerman et al. (2023) found comparable results, with a discrimination index of 0.23 and a difficulty index of 0.71 for 25 MCQs. These findings align with the current study, highlighting consistent patterns of difficulty and discrimination that meet or exceed acceptable standards for educational assessments (Zuckerman et al., 2023).

The Anderson-Darling test provided further evidence of significant differences between the two exams, with a test statistic of 26.84 (p < 0.001). This result confirms that the distributions of grades in 2023 and 2024 were distinct, reflecting changes in the structure and difficulty of the exams. The more varied question difficulty in 2024 is likely responsible for the observed shift, indicating that the AI-assisted question generation may have contributed to improved assessment quality. By introducing a broader range of question difficulties, the 2024 exam offered a more comprehensive evaluation of student learning, moving away from the grade inflation observed in 2023.

Previous studies have reported that ChatGPT-generated MCQs exhibit inaccuracies ranging from 16% to 60%, emphasizing the importance of thoroughly reviewing these questions before using them in medical education assessments (Kiyak and Emekli, 2024). Although ChatGPT-generated MCQs have achieved the goal of reducing grade inflation, previous literature highlights the benefit of reducing the time required to create MCQs from 30 to 60 min to just 5–15 min (Zuckerman et al., 2023). Another study found that creating case-based MCQs with ChatGPT took 20 min and 25 s, compared to 211 min and 33 s for humans (Cheung et al., 2023). However, the current study did not assess the time required for generating questions; instead, it confirmed the necessity of carefully evaluating AI-generated MCQs to ensure their accuracy and suitability before incorporating them into exams. Despite the time-saving advantages, these benefits should not outweigh the potential risks of academic misconduct, overreliance on AI, and concerns about the accuracy and reliability of the generated questions (Benítez et al., 2024).

Future studies should focus on longitudinally tracking students’ performance and learning outcomes over multiple years to evaluate whether AI-generated assessments have a sustained impact on educational rigor and grade distribution.

We recommend establishing a structured protocol for human oversight when using AI-generated questions to enhance their reliability and suitability. This protocol should include clear criteria for evaluating question accuracy, a standardized process for resolving discrepancies between AI outputs and human judgment, and collaboration between AI developers and subject matter experts. Such measures would help ensure the quality and appropriateness of AI-generated assessments, particularly in disciplines with complex terminologies or nuanced content like Pharmacology.

Despite these promising results, several limitations should be acknowledged. Although the AI-generated questions appeared to improve the quality of the 2024 exam, the study relied on a limited number of MCQs and focused on a single course. The findings may not be generalizable to other disciplines or educational contexts without further validation. Additionally, while the reduction in skewness and increase in reliability are promising, future studies should investigate the long-term impact of AI-generated questions on student learning outcomes and performance in subsequent courses.

## 5 Conclusion

In conclusion, this study demonstrates that incorporating AI-generated MCQs can enhance the quality of assessments by reducing grade inflation and improving exam reliability. The findings suggest that AI tools such as ChatGPT can assist educators in developing more balanced exams, offering a range of easy, moderate, and challenging questions that better differentiate student performance. The 2024 exam, designed with the aid of AI, exhibited improved reliability and a more balanced grade distribution compared to 2023. These results highlight the potential of AI-assisted assessments to promote fairness and integrity in higher education. Future research should explore the broader application of AI-generated assessments across different disciplines or courses and evaluate their impact on long-term learning outcomes.

## Data Availability

The raw data supporting the conclusions of this article will be made available by the authors, without undue reservation.
